# Seed Protein Content Estimation with Bench-Top Hyperspectral Imaging and Attentive Convolutional Neural Network Models

**DOI:** 10.3390/s25020303

**Published:** 2025-01-07

**Authors:** Imran Said, Vasit Sagan, Kyle T. Peterson, Haireti Alifu, Abuduwanli Maiwulanjiang, Abby Stylianou, Omar Al Akkad, Supria Sarkar, Noor Al Shakarji

**Affiliations:** 1 Department of Computer Science, Saint Louis University, Saint Louis, MO 63104, USA; imran.said@slu.edu (I.S.);; 2 Department of Earth, Environment and Geospatial Sciences, Saint Louis University, Saint Louis, MO 63108, USAomar.alakkad@taylorgeospatial.org (O.A.A.); supria.sarkar@slu.edu (S.S.);; 3 Taylor Geospatial Institute, Saint Louis, MO 63108, USA; 4 Bayer Crop Science, 800 N Lindbergh Blvd, Creve Coeur, MO 63141, USA

**Keywords:** hyperspectral imaging, seed composition estimation, machine learning, 3D CNN modeling, attentive models

## Abstract

Wheat is a globally cultivated cereal crop with substantial protein content present in its seeds. This research aimed to develop robust methods for predicting seed protein concentration in wheat seeds using bench-top hyperspectral imaging in the visible, near-infrared (VNIR), and shortwave infrared (SWIR) regions. To fully utilize the spectral and texture features of the full VNIR and SWIR spectral domains, a computer-vision-aided image co-registration methodology was implemented to seamlessly align the VNIR and SWIR bands. Sensitivity analyses were also conducted to identify the most sensitive bands for seed protein estimation. Convolutional neural networks (CNNs) with attention mechanisms were proposed along with traditional machine learning models based on feature engineering including Random Forest (RF) and Support Vector Machine (SVM) regression for comparative analysis. Additionally, the CNN classification approach was used to estimate low, medium, and high protein concentrations because this type of classification is more applicable for breeding efforts. Our results showed that the proposed CNN with attention mechanisms predicted wheat protein content with R^2^ values of 0.70 and 0.65 for ventral and dorsal seed orientations, respectively. Although, the R^2^ of the CNN approach was lower than of the best performing feature-based method, RF (R^2^ of 0.77), end-to-end prediction capabilities with CNN hold great promise for the automation of wheat protein estimation for breeding. The CNN model achieved better classification of protein concentrations between low, medium, and high protein contents, with an R^2^ of 0.82. This study’s findings highlight the significant potential of hyperspectral imaging and machine learning techniques for advancing precision breeding practices, optimizing seed sorting processes, and enabling targeted agricultural input applications.

## 1. Introduction

Wheat (*Triticum aestivum* L.) is a cereal which is cultivated extensively across the world, with millions of tons of grains being harvested annually, and serves as a staple for many communities worldwide [1]. A significant portion of the total mass of wheat seeds consists of proteins, which serve several different functions. Proteins in durum wheat are not sufficient as a primary provider of essential amino acids [2], mostly due to the less anabolic nature of wheat proteins compared to animal proteins due to their lower comparative digestibility [3]. This low digestibility is compounded further by cooking processes such as baking [4]. However, when consumed alongside [3] or infused with other non-plant-based proteins [2], wheat products have been found to complement these primary protein sources well. Additionally, processes which have been introduced to solve their lower digestibility have made wheat proteins a great source of protein for senior consumers [5]. The proteins in wheat seeds also serve another crucial role: structural. In particular, the gluten proteins, which make up 70–80% of total grain proteins, form a mesh in which starch granules are deposited [6] and are foundational to the distinctive physical properties of dough and the bread-making quality of wheat. This invariably means that the gluten proteins have an influence on the technological performance of different wheat varieties in food production and on the final price of wheat [7]. This calls for the initiation and encouragement of processes that allow analysis and documentation of the qualitative and quantitative characteristics of protein in wheat seed kernels. Furthermore, these processes need to be non-destructive and efficient to accelerate selection processes in breeding programs, while also preserving valuable genetic material for subsequent breeding cycles.

Hyperspectral imaging (HSI) plays an important role in estimating seed composition to automate the process, as the distinct chemical composition of the sample contributes to the relative intensity in absorption, reflection, and transmission of incident radiation [8,9]. This can accelerate seed inspection and breeding efforts as chemical analysis methods often require large, ground samples and involve destructive analysis procedures that can be costly and time-consuming. HSI entails the use of specialized sensors to scan subjects across specific continuous wavelengths of the electromagnetic spectrum, creating three-dimensional datasets composed of two spatial dimensions, and a spectral dimension representing different reflectance values for each pixel of the image [10,11].

HSI has been used to predict seed composition traits of individual kernels, including fatty acids [12], oil [13], moisture [14], and proteins [15,16], enabling the rapid screening of large numbers of samples for the selection of seeds with desirable composition traits [11,13]. Protein content prediction using NIR and HSI for wheat seeds has been reported by several publications with spectral features extracted from imagery [11,17,18,19]. To the best of our knowledge, there have been no studies that (1) utilized both VNIR and SWIR images as direct input for end-to-end seed composition estimation and (2) investigated the contribution of ventral and dorsal seed orientations for wheat seed protein determination.

This can be attributed to previous studies showing that the NIR region contains the most informative wavelengths for protein content determination, with one study establishing a high vs. low protein content discriminatory technique based on NIR [20], whereas [21] applied PLSR on NIR data with different scatter correction techniques to predict protein content in wheat seeds. Among these studies, Caporaso et al. [11,15] found the wavelength of 1918 nm to be most informative, followed by 2008 nm, 2062 nm, and 2272 nm. Delwiche et al. [22] found wavelengths 1100 nm and 1398 nm to produce the lowest overall model error, while Liu et al. [23] identified five wavelengths between 1980 nm and 2350 nm that correlate strongest with gluten proteins. This region covers numerous overtones and combination bands of molecular vibrations, particularly those in the amide functional groups (N-H and C=O bonds), the chemical components that form the building blocks of proteins and which absorb more NIR light [8,24]. The first overtone of N-H stretching modes typically occur in the 1400–1600 nm range, while the combination bands involving N-H and C=O groups typically occur within the 2000–2300 nm range [25].

In this paper, we present a new approach to protein content estimation that combines attentive vision models and hyperspectral images of wheat seed scans. We also present the calibration and data preprocessing methods necessary to make this kind of modeling possible.

The objectives of this paper are as follows:

1. Investigating the potential of hyperspectral imaging to predict protein content values for wheat seeds;
2. Classifying wheat seeds according to protein content;
3. Determining optimal wavelengths that are the most informative to the prediction of protein content in seeds;
4. Determining the effect of the orientation of wheat kernels on the predictive ability of machine learning models.

## 2. Materials and Methods

### 2.1. Wheat Seed Sample Description

The wheat seed samples were provided in two batches by the Crop Science division of Bayer (Creve Coeur, MO, USA). The first batch consisted of individual seeds sorted according to shared genotypical traits. In total, we had 9 genotype classes, whose frequencies are listed in Table 1. To allow for a parallel comparison of ventral and dorsal scans of each seed sample, they were retained separately in a tray with fixed labels before and after scanning. 

The second batch of seeds was grouped in samples based on their protein content obtained through NIR spectroscopy scans. These were later divided into smaller subsamples for a total of 188 subsamples, with at least 60 seeds per subsample, which were scanned separately, and then protein content values for each subsample were obtained separately.

### 2.2. HySpex Hyperspectral Imaging System

**Figure 1:**
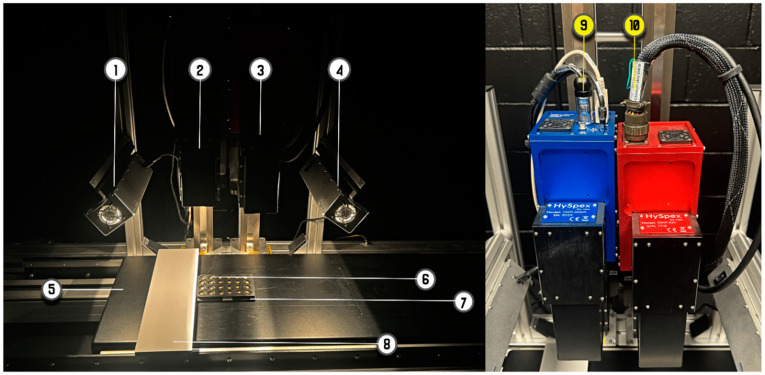
HySpex bench-top scanning machine used in this study. Components as labeled in order: (1) lamp serving as illumination source for VNIR scanner, (2) HySpex VNIR-3000N sensor, (3) HySpex SWIR-640 sensor, (4) SWIR lamp, (5) scanning stage, (6) wheat seed sample, (7) tray holding the seed samples, (8) reflectance panel, (9) VNIR power source, and (10) SWIR power source.

The scans for the seeds were acquired using two hyperspectral imaging sensors of the HySpex brand manufactured by Norsk Elektro Optikk (Oslo, Norway), mounted on an indoor scanning stage as illustrated in Figure 1. The first system, the HySpex VNIR-3000 N [26], has a pixel size of 3.45 μm, which ensures that even narrow band features are resolved with great precision. It also features 3000 spatial pixels and 300 bands covering wavelengths from 400 nm to 1000 nm. The second imaging system, the HySpex SWIR-640 [27], consists of a Mercury Cadmium Telluride (MCT) sensor, which offers a spatial resolution of 32 μm and features a cryogenic cooler that cools the focal plane array to 150 K to minimize background noise and offer exceptional signal-to-noise ratio levels. The SWIR sensor has 362 bands from wavelengths of 960 nm to 2500 nm. The spectral resolutions of the VNIR and SWIR regions are 1.2 nm and 1.7 nm, respectively.

For both systems, the source of illumination of the samples consisted of two custom-made lamps which were manually adjusted to focus the illumination to a line overlapping with each camera’s field of view [28]. They cover a broad spectral range from 400 nm to 2500 nm, which sufficiently covers the spectral range of both cameras, and are powered by a 12 V DC supply. The scanners capture the image line by line (line-scanning) while the specimen on the stage is passed across their fields of view in a technique known as pushbroom scanning. A 50% spectralon reflectance panel was employed for calibration.

The combination of mechanisms on the imaging system to reduce signal-to-noise ratio levels ensures that less time is spent in calibrating the images during the post-processing stage. The high spatial resolutions of the scanners ensures that we capture as much textural detail as possible, while the precise spectral resolution and broad wavelength coverage allow for the accurate detection of spectral signatures associated with protein and other seed components, enabling more comprehensive chemical and physical analyses.

### 2.3. Hyperspectral Scanning

The seeds were scanned in two batches. The first batch was scanned over a period of two months, between May and June of 2023, and involved around 2398 samples. Single seeds were placed on circular troughs on the scanning plate, spanning 4 wide and 6 lengthwise to make a total of 24 seeds scanned at a time. The seeds were all scanned with the crease facing upwards (ventral view) and then a second time with the crease facing downwards (dorsal view). The seeds were then immediately replaced in their storage trays and the lamps were turned off once the scanning was completed to prevent excessive moisture loss. The second batch of seeds was scanned in November 2023 over a period of three weeks and involved around 11,000 samples. This time, the seeds were placed randomly on the scanning stage, with heterogeneous orientations of the seeds available with each scan.

Given that there is no commercially available software to co-register VNIR and SWIR spectral datasets, we implemented a novel approach by introducing fiducial markers within the cameras’ field of view. These tie pointswere placed on a specialized paper substrate and served as invariant reference points across both spectral ranges. Our team developed a proprietary co-registration algorithm that leverages these fiducial markers to achieve high-precision spatial alignment between the VNIR and SWIR hyperspectral data cubes.

### 2.4. Reference Data Collection

The protein content of the seeds was obtained after scanning of the images via Dumas combustion chemical analysis. Dumas has been used in previous studies to obtain nitrogen [29] and protein content [30,31] in cereals and other foodstuffs and has been documented as outperforming other chemical analysis methods for most applications [32,33].

The process is based on the concept of the extraction of nitrogen oxides from the materials of study through rapid combustion of the material in a high-heat chamber, followed by a reduction of the nitrogen oxides to nitrogen gas (N2). The final step involves the quantification of N2 using thermal conductivity.

From the chemical analysis, we were able to obtain percentages of protein mass against total seed mass with a range of 11.96%, with a minimum of 7.69% and a maximum value of 19.65%. The rest of the statistics for each orientation are summarized in Table 2.

We defined protein content classification labels for each of the seeds according to the amount of protein content, with anything below 10% labeled as low, 10–14% as medium, and above 14% as high.

### 2.5. Workflow

**Figure 2:**
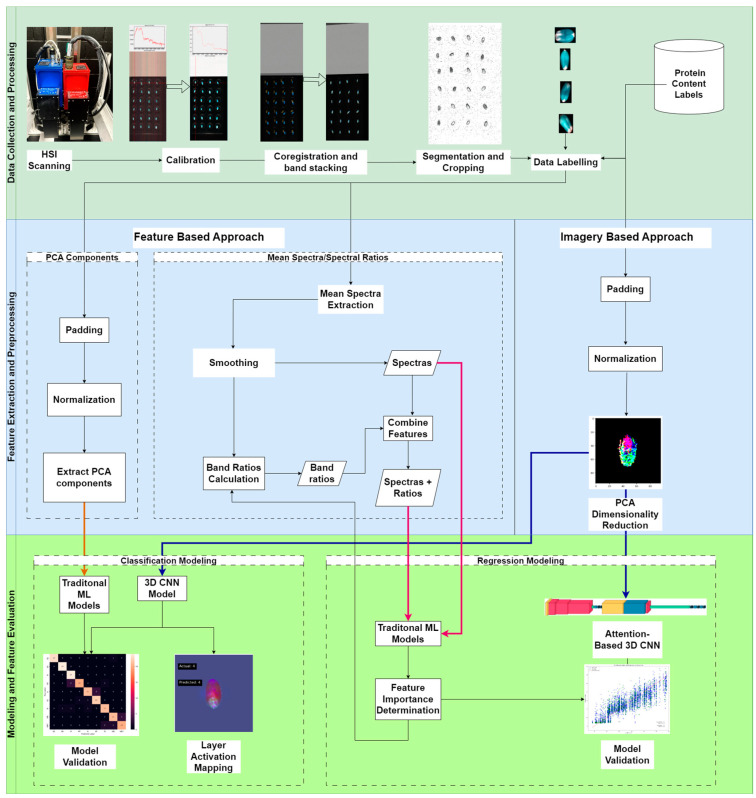
Overview of the workflow for our pipelines from data collection all the way to model validation.

An overview of the methods applied within our study is presented in this section and summarized in Figure 2. The three main stages of the processes include the data collection and processing step, followed by the feature extraction and image preprocessing stage, which is further divided into feature-based approaches and image-based approaches, and finally the modeling and feature evaluation stage.

### 2.6. Radiometric Calibration

**Figure 3:**
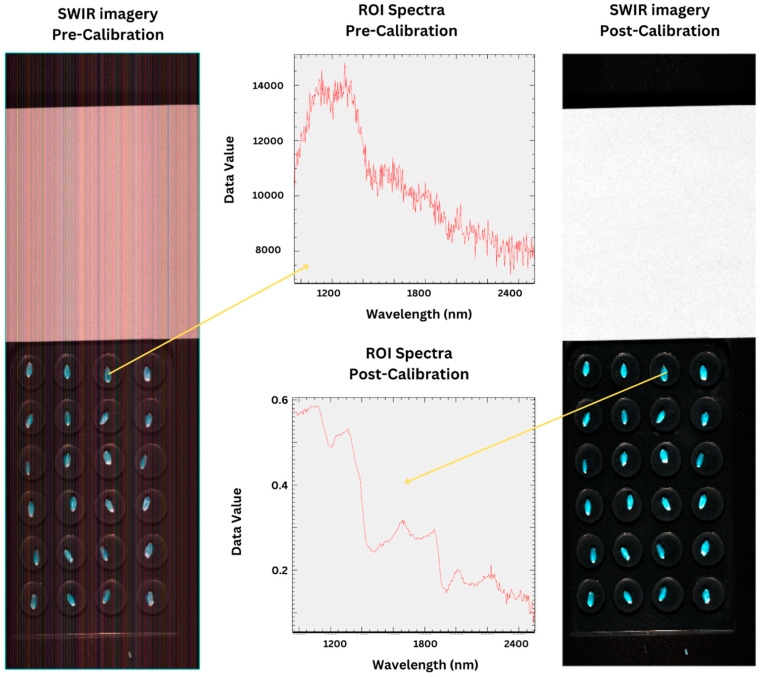
A comparison of the imagery and spectral profiles of the scan scenes (confined to SWIR scans) to highlight the effect of radiometric calibration.

Wheat seeds have intrinsically anisotropic reflection properties caused by their uneven surfaces and variable color depth characteristics, which can significantly influence the spectral information captured by hyperspectral imaging systems by causing drastic variability in the data, depending on incident light and sensor viewing angles. The size of a single wheat kernel (about 3 mm along/1.25 mm across) is often smaller than the irradiated diameter (spot size) of the sensor, causing a significant presence of scattering of the reflected signal from the edges of the kernel [34] which contributes to the overall deterioration of signal quality as demonstrated in the pre-calibration spectral profile of the seed in Figure 3. This presents a challenge for fidelity of the data and necessitates the application of reflectance correction methodologies to normalize the hyperspectral data and therefore ensure that it accurately represents the seeds’ true spectral properties across different samples and scanning sessions. For hyperspectral analysis of wheat seeds, the fidelity of the data has a direct influence on the reliable estimation of biophysical properties and can prevent inconsistencies in comparative studies across different datasets.

Previous studies have successfully managed to address the reflectance correction challenge of hyperspectral imagery through various methods, including absolute radiometric correction [35], the empirical line method [36], flat field correction [37,38], and Bidirectional Reflectance Distribution Function (BRDF) correction [39,40], among others. Our methodology adopts a reflectance correction approach tailored to bench-top hyperspectral scanning systems and is facilitated by the standard calibration procedure provided by the instrument manufacturer, Norsk Elektro Optikk (NEO). This calibration workflow consisted of the following steps [41]:

1. Background signal subtraction: The sensor’s dark current response (*Sbg*), measured when no light is supplied to the sensor, is subtracted from the raw signal (*Sraw*) to correct for background electronic noise. For pixel (*i*, *j*) this is summarized as
  (1) $$S_{i,j}=S_{raw,i,j}-S_{bg,i,j}$$
2. Radiometric response: A bright scan using the reflectance panel referenced above, with a known reflectance value $L_{ref}$, is used to obtain a signal $S_{bright,i,j}$ which is then used to calculate the radiometric response $R_{i,j}$ using the following formula:
  (2) $$R_{i,j}=\frac{S_{bright,i,j}-S_{bg,i,j}}{L_{ref}}$$
3. Radiometric calibration: The background-subtracted signal is converted to spectral radiance L in units $\left(W/sr\cdot m^{2}\cdot nm\right)$ incident at the sensor’s entrance pupil using the radiometric response matrix R, calculated above, and the integration time $t_{int}$, as per the following equation:
  (3) $$L_{i,j}(\lambda_{i,j})=S_{i,j}\cdot \frac{1}{R_{i,j}\cdot t_{int}}$$

### 2.7. Co-Registration of Images

To take advantage of both spectral and textural features of our subjects, the VNIR and SWIR images need to be combined/stacked correctly and efficiently. To do so, we need to map coordinates of our fine-resolution VNIR (with spatial resolution of 32 μm) scans to our coarse-resolution SWIR (spatial resolution of 3.45 μm) scans. A common approach to achieving this involves resampling the VNIR image by scaling the dimensions of the VNIR (target) using an interpolation function interp like INTER_AREA [42]. If we assume that $h_{VNIR}$ and $w_{VNIR}$ represent the height and width of VNIR image, respectively, and $h_{SWIR}$ and $w_{SWIR}$ represent the height and width of the SWIR image, respectively, ${scale}_{h}$ and ${scale}_{w}$ represent the scaling factors height and width, respectively, which can be calculated as follows:

(4) $${scale}_{h}=\frac{h_{VNIR}}{h_{SWIR}},\;\;{scale}_{w}=\frac{w_{VNIR}}{w_{SWIR}}$$

**Figure 4:**
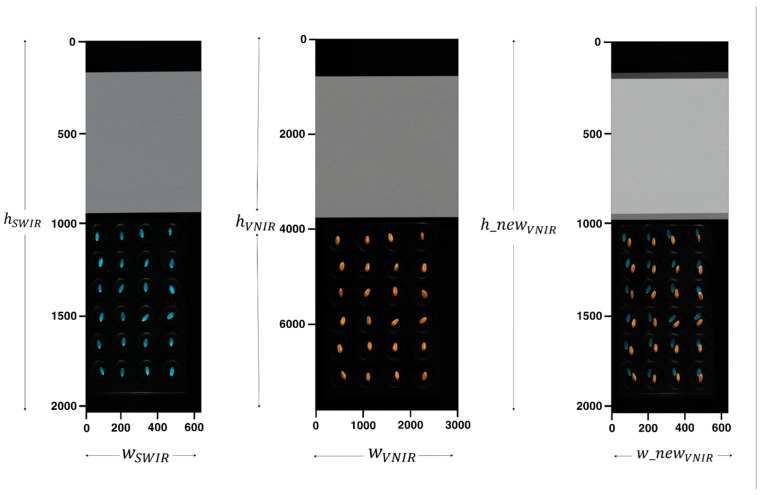
Validation of the need for co-registration. The image on the right shows the result of stacking SWIR and VNIR images after resampling of the VNIR image. Here, the new dimensions of VNIR, ${w\_new}_{VNIR}=round$($\frac{w_{VNIR}}{{scale}_{w}}$) and ${h\_new}_{VNIR}=round$($\frac{h_{VNIR}}{{scale}_{h}}$), exactly match the dimensions of the corresponding SWIR image, $w_{SWIR}$ and $h_{SWIR}$, respectively. However, the invariance of the method to the horizontal and vertical translations of the image reveals a significant shift between the pixels of the stacked images and will result in more errors.

However, this method demonstrates invariance to both horizontal and vertical translations, as shown in Figure 4. Furthermore, the differences in sensor height resulted in a noticeable horizontal shift in the VNIR images relative to the SWIR images. In this case, differences in scale, rotation, and translation between the source and target images necessitate the use of co-registration [43,44]. In our study, we needed to address two of these three: scale and translation.

Solutions from the public domains that we tested did not produce results within acceptable margins of errors for various reasons—for instance, applying traditional co-registration methods using feature detection and matching, such as Scale-Invariant Feature Transformation or (SIFT) [45] and Speeded-Up Robust Features (or SURF) [46]—and optical flow was hampered by (a) differences in vertical positioning of the scanners, resulting in differences in amounts of detail captured by the different sensors; (b) the differences in spectral ranges captured by the two sensors (400–1000 nm for VNIR and 960–2500 nm for SWIR), which caused the reflectance characteristics of the imaged scene elements to differ significantly; (c) differences in intensity values for the same spectral bands overlapping across the two sensors; and (d) the presence of numerous visually similar artifacts within the imaged scene (in this case, the seeds), causing ambiguity due to the repetitive nature of seed edges and creases.

**Figure 5:**
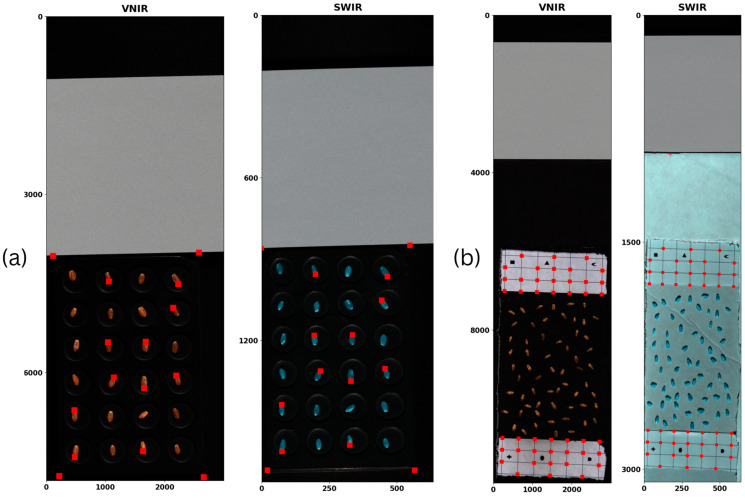
Tie points used to calculate homography for each batch of seed scans. (**a**) The red dots indicate tie points identified manually on the seeds and the background. These were meticulously picked and curated until the lowest error was achieved. (**b**) The second batch of seeds was scanned with tie points placed alongside the samples to ensure minimal manual identification of tie points.

A customized approach to co-registration of our images was therefore necessary. For the first batch of images, fiducial tie point markers were identified manually, illustrated in Figure 5a, using ENVI software. Meanwhile, for the second batch, fiducial markers were placed during the scanning process as mentioned earlier in the text as shown in Figure 5b.

Corresponding coordinates of the tie points from both images were then used for calculation of a homography matrix which maps the rest of the target image’s pixels to their corresponding positions in the reference image. Homography describes the transformation of one point from an image to its corresponding point in another image with the assumption that the scene is flat, and the transformation can be estimated by a planar projective transformation. It can be represented in formula as

(5) $$H=\left[\begin{array}{c} h_{11\;\;}\\ h_{21\;\;}\\ h_{31\;\;} \end{array}\begin{array}{c} {\;h}_{12}\;\;\\ {\;h}_{22}\;\;\\ \;h_{32}\;\; \end{array}\begin{array}{c} h_{13}\\ h_{23}\\ h_{33} \end{array}\right]$$

where H is our projective transformation 3 × 3 matrix with the elements of the matrix, i.e., $\left(h_{11},\;h_{12},\ldots ,\;h_{33}\right)$, being determined by the correspondence between several matched points in the two images, typically at least four of these. In our case, we obtained a minimum of 10 points and at most 17 matched points for our first scan batch and 32 for the second batch of scanned seeds, mapping original points x and y onto their corresponding coordinates $x^{\prime }$ and $y^{\prime }$ on the second image, as shown in the formula below:

(6) $$\left[\begin{array}{c} x^{\prime }\\ y^{\prime }\\ w^{\prime } \end{array}\right]=H\left[\begin{array}{c} x\\ y\\ 1 \end{array}\right]$$

Here, $\left(x^{\prime },y^{\prime },w^{\prime }\right)$ are the homogeneous coordinates. To convert these back to Cartesian coordinates, the homogenous coordinates are divided by $w^{\prime }$.

(7) $$x^{\prime }=\frac{h_{11}x+h_{12}y+h_{13}}{h_{31}x+h_{32}y+h_{33}},\;y^{\prime }=\frac{h_{21}x+h_{22}y+h_{23}}{h_{31}x+h_{32}y+h_{33}}$$

The transformation is not merely linear but projective as well, meaning it accounts for the perspective changes due to the angle of view. The fact that our sensors capture images from different heights and slightly different angles therefore makes the choice of using homography transformation the most viable one.

To calculate the homography matrices for the images, we used the robust RANSAC (Random Sampling Consensus) algorithm provided by the open-source library OpenCV [47] with a threshold of 7.0. The resulting transformation matrix was then applied to the target image using the warpPerspective [42] function from the same library, which warps the dimensions of the target image along the projective transformation matrix provided. To quantitatively assess the precision of our alignment method, we employed the mean squared error (MSE) and root mean squared error (RMSE) as metrics.

### 2.8. Background Segmentation, Cropping, and Labeling

Spurious signals from the pixels of the background tray and the reflectance panel will affect the overall mean reflectance spectra of our seed scans and therefore need to be removed. This will allow the predictive models to focus attention purely on the signals from the seed scans. Similar studies have removed spurious signals caused by background material in hyperspectral scans of vegetation using a rule-based approach [39,48], as well as removing soil background from maize [49] using vegetation indices, and also in a different study has been applied to distinguish Petri dish salmon samples using Otsu thresholding [50]. Specifically for bench-top scans, other researchers applied a combination of Otsu thresholding and morphological approaches to segment rice granules from a dark background [51,52].

**Figure 6:**
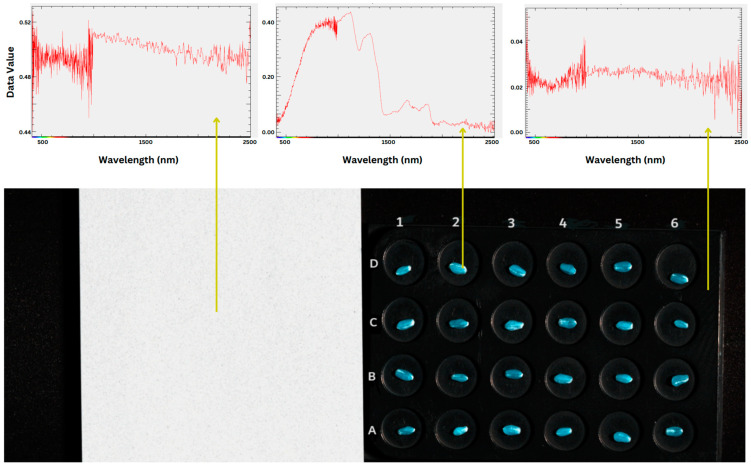
Differences in spectral profiles of the seeds from those of the tray and reflectance panel allow for creation of a rules-based segmentation mechanism to allow separation of seeds from the background. As per earlier explanation, the first batch of seeds were aligned in rows 4-seed-wide labeled A–D and 6-seed-long labeled 1–6.

For the first step of segmentation, a rule-based masking algorithm was applied, taking advantage of the intrinsic differences in reflectance properties between our wheat samples and the background materials, combined with morphological operations to retain seed pixels and remove noise. This approach is based on the distinct spectral signatures exhibited by the seeds, background tray, and reflectance panel, as evident from the spectral profiles shown in Figure 6. The algorithm utilizes reflectance values at specific wavelengths—410 nm, 456 nm, 553 nm, 654 nm, and 852 nm bands—which capture the most significant variations between the seeds and the background materials, abbreviated as B1, B2, G, R, and N, respectively, in the formula below:

(8) $$p_{i}=\left\{\begin{array}{c} 1,\;for\;condition1\;and\;condition2\;and\;condition3\;and\;condition4\;and\;condition5\\ \;0,\;for\;condition6\;and\;condition7\;and\;condition8\;and\;condition9\;and\;condition10 \end{array}\right.$$

$$\begin{array}{c} {condition1=p}_{i,B1}<p_{i,B2}\\ condition2=0.7*p_{i,B1}\leq p_{i,R}\geq 12.5*p_{i,B1}\\ condition3=1.2*p_{i,B1}\leq p_{i,N}\geq 15*p_{i,B1}\\ condition4=p_{i,B2}<p_{i,G}\\ condition5=1.2*p_{i,B2}<p_{i,G}\\ {condition6=p}_{i,B1}>p_{i,B2}\\ condition7=0.7*p_{i,B1}>p_{i,R}<12.5*p_{i,B1}\\ condition8=1.2*p_{i,B1}>p_{i,N}<15*p_{i,B1}\\ {condition9=p}_{i,B2}>p_{i,G}\\ condition10=1.2*p_{i,B2}>p_{i,G} \end{array}$$

The rule defines the expected ratio of values of a seed pixel between pairs of bands that distinguishes it from non-seed pixels and was determined experimentally by comparing initial spectral profiles of regions of interest on seed pixels against those of the background pixels. If a pixel satisfies all the defined conditions, then the pixel is considered a seed pixel and retained (assigned a value of 1). Otherwise, the pixel is considered part of the background or shadow and discarded (assigned a value of 0).

**Figure 7:**
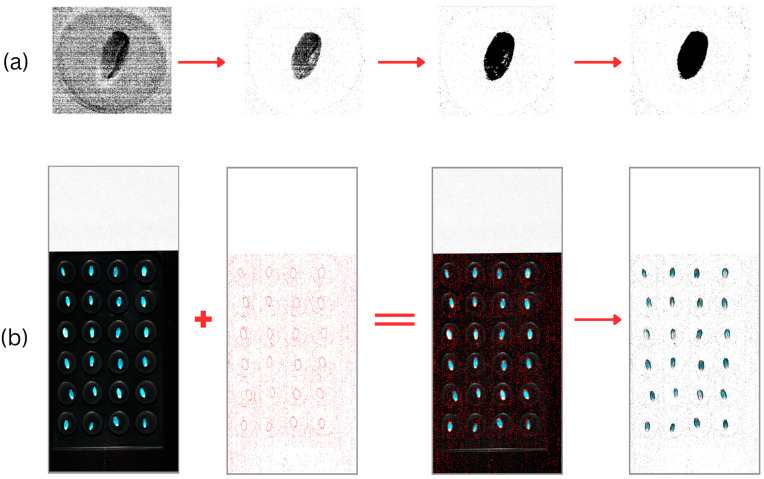
Image segmentation process, showing the process of obtaining and applying masks to segment the seeds from the background. This was achievable due to the spectral differences and color differences between the seeds and background material. (**a**) A zoomed-in single image highlighting the refinement of criteria for the rule-based segmentation approach which, results in the formation of ‘holes’ within the seed pixel regions and the subsequent result of using flood fill to help fill the ‘holes’. (**b**) The application of the generated mask to extract seeds from the background.

For the second step, morphological operations, a technique widely used in image processing for noise removal and boundary smoothing [53], were employed to address persisting background pixels and missed seed pixels, as illustrated in the second and third images of the provided sequence in Figure 7a. A morphological opening operation was performed using a 3 × 3 structuring element kernel using OpenCV’s provided method, morphologyEx [54].

Since the full seed images would be used to train a predictive vision model, all the pixels within the established boundary of the seed mask needed to be retained. As such, for the third step, a flood-fill algorithm was implemented, a region-based approach commonly used for image segmentation [55,56,57]. Starting from the top-left corner (0, 0) of the image, the flood-fill operation provided by OpenCV [58] effectively filled the background region with a new value (2), while preserving the seed pixels (value 1) and any remaining noise pixels (value 0).

In the post-processing stage, any remaining “noise” pixels which were not filled by the flood-fill operation were addressed by assigning them to the seed class (value 1), as we can safely assume these to be “holes” within the seed pixel regions, as shown in Figure 7a. The filled background region was relabeled as class 0. Finally, the border added during the flood-fill operation was removed to obtain the final segmented image, containing only the seed pixels and the background.

To crop individual seeds from the image, an OpenCV-provided methodology was used, which specified three main steps. The first involves retrieving a three-band image consisting of the red, green, and blue channels. In our study, a single band from each section of the visual range of wavelengths was retrieved, including red (642 nm), green (546 nm), and blue (460 nm) bands, to compose our three-channel image. This image then had to be converted to grayscale, and the result was transformed into a binary format to distinguish the seeds from the background.

**Figure 8:**
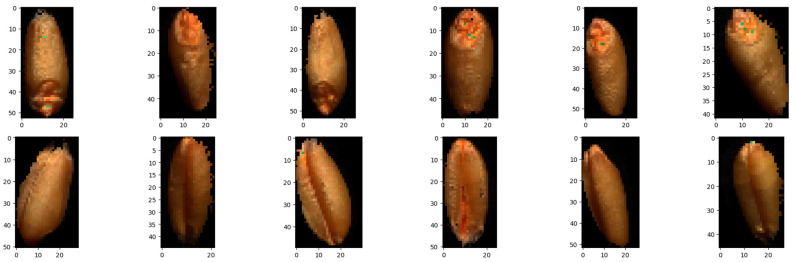
Sample of final cropped single-seed images, with the top row showing the seeds with the crease down (**top** view) and the bottom row showing the seeds with the crease up (**bottom** view).

Following this, a connected-component analysis using OpenCV’s connectedComponentsWithStats method [59] was applied to identify and isolate discrete seed regions, achieved by filtering for regions with areas that satisfied the following threshold: 250 < area < 1500. The isolated-component areas were then labeled according to a scheme facilitated by the spatial ordering of the identified seeds based on their original positions in a grid-like structure. The decision to take this systematic approach to labeling of the seeds was to enable us to correlate each seed scan to its correct protein content labels. This approach would also enable a comparative analysis against and potential fusion with other modes of data captured of the same seeds. A sample of the final single seed images is illustrated in Figure 8.

In summary, for the segmentation portion of the workflow, we applied a combination of a rule-based masking algorithm, morphological operations, a flood-fill algorithm, and post-processing steps to isolate seed pixels from the background. For the subsequent cropping phase, we extracted a three-band image, converted it to grayscale and binary format, performed connected-component analysis, and applied area-based filtering to isolate individual seeds.

### 2.9. Comparison of Average Spectra per Protein Content

**Figure 9:**
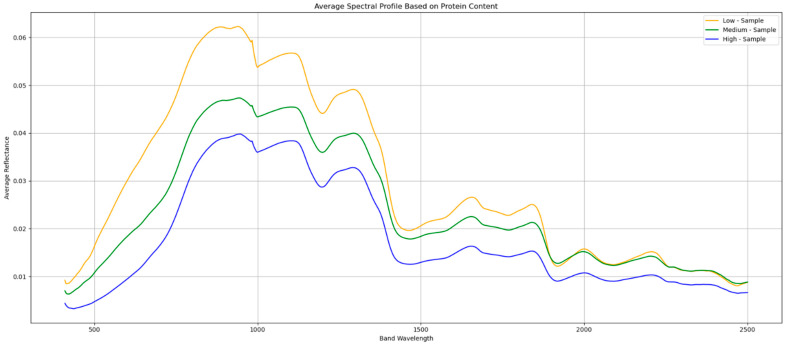
Comparison of average spectra of wheat seed based on the protein content.

We were able to delineate a correlation between the amount of protein content in the seeds and the intensity of their reflectivity, as demonstrated in the graph in Figure 9, with seeds of higher protein content demonstrating lower reflectivity properties across all the bands, while lower-protein-content seeds indicated the highest intensity but also showed the largest dips at wavelengths 1400–1500 nm and 1900–2000 nm, which could suggest a relatively higher moisture content in the seeds with the lowest protein content, as these bands are the most affected by moisture [60].

### 2.10. Feature-Based Machine Learning for Protein Content Estimation

The average spectra of each seed scan were calculated and stored for further analysis. In HSI applications, signal smoothing is often necessitated by the presence of noise caused by the scanning instruments [61,62]. We discovered that the signal was mostly free of noise except for the last few bands. We therefore applied minimally intrusive smoothing on the spectra using the Savitzky–Golay [63] method provided by the SciPy library [64], with a window of 7 and polyorder of 3.

Spectral indices have been used previously in HSI studies to successfully predict different properties of numerous study subjects, including soil properties [65,66] and crop yield [67,68]. While there are currently no spectral indices developed specifically for bench-top scans of seeds, we attempted a simple band ratio approach in attempt to improve the predictive capabilities of our models. Given a sample i and for each unique pair of bands j and k (where j<k), this approach can be summarized as

(9) $$R_{ijk}=\frac{X_{ij}}{X_{ik}}$$

where $R_{ijk}$ represents the ratio of the spectral values between bands j and k for sample i, j iterates from 1 to the total number of bands, and k iterates from j+1 to the total number of bands.

These data were then normalized to ensure that all features contributed proportionately to the objective function, preventing those with larger scales from dominating the optimization process of our predictive models [69]. Furthermore, by scaling our features to a common scale, normalization mitigates the issues of convergences in algorithms sensitive to the scale of the input data, such as logistic regression and Support Vector Machines [70].

We processed 5057 samples for the top orientation and 4746 for the bottom orientation. These samples were then split into train, test, and validation, with a test to train ratio of 3:1 and a validation to train ratio of 4:1. This yielded train, test, and validation datasets of 3034, 1264, and 759, respectively, for top orientation and 2847, 1187, and 712, respectively, for bottom orientation.

To extract the most informative bands for the regression modeling, we performed permutation feature importance analysis on each model and extracted the top 100 wavelengths. We then calculated the ratios of these bands, combined them with the original 100 bands, and trained a fresh set of models from scratch. This essentially yielded three different datasets, as summarized in Table 3.

#### 2.10.1. Random Forest Regression

Random Forest is an ensemble learning method which operates by constructing multiple decision trees and harmonizes the outputs of the individual trees to produce the final result [71]. At the individual tree level, random splitting of the features occurs at each node, resulting in increased variation among the trees and thus improving the robustness of the final model [71]. This is supplemented by a technique called bagging or bootstrap aggregating, a technique that involves building each tree on a bootstrap sample of the data, which helps reduce overfitting [72]. Random Forest can handle high-dimension spaces as well as large sample sets very effectively too, making it a good choice for our regression task.

#### 2.10.2. Support Vector Regression

Support vector regression is a type of Support Vector Machine used for regression tasks but based on the same principles used for SVM classification, which is to find a best-fit line within a threshold value ε where distances from the actual targets $y_{i}$ to the line are minimized and at as many as possible points lie within the ε-tube without penalty [73]. To determine this line, a combination of the points closest to the fit (support vectors) and a regularization parameter, C, are used to balance between minimizing model complexity and achieving a lower error on the training data. To solve nonlinear regression tasks, SVR uses kernel functions to map input data into higher-dimensional space where a linear regression can be fitted [74]. SVR is known for its strong performance in smaller datasets and its capability to manage high-dimensional spaces and has shown promising results in previous similar research [75,76].

### 2.11. Image-Based CNN Approaches

#### 2.11.1. Data Preprocessing Methods

Data need to be scaled and normalized for CNN training pipelines as they facilitate faster convergence of the model [77] and improve the stability of the neural network [78]. Meanwhile, other research shows that normalizing within the neural network architecture itself through batch normalization can facilitate a more stable gradient flow and acceleration of the training process [78], is instrumental in preventing vanishing and exploding gradients [79], and in some cases has been shown to have a regularization effect that can prevent overfitting in neural networks [80]. For our study, we applied minmax normalization.

The final co-registered images consist of 662 contiguous bands over a wavelength range between 412 and 2500 nm. While this high dimensionality facilitates capturing intricate spectral profiles, it unfortunately suffers from a few intractable limitations, including the ‘curse of dimensionality’ phenomenon, where the complexity of data analysis tasks grows exponentially with increases in dimensionality [81]; data sparsity in higher dimensional spaces [82]; and increasing the effects of noise and irrelevant features [83]. We therefore applied dimensionality reduction to our data using PCA (Principal Component Analysis), a commonly used dimensionality reduction method for HSI applications [62,84,85].

Starting with our four-dimensional tensor with shape (*H*, *W*, *C*), where *H* and *W* are our spatial dimensions for each seed scan and *C* is the number of channels or spectral bands, we transform individual seed scans into a 2D vector of shape (*H* × *W*, *C*), thus presenting each pixel as a distinct observation within the channel space. Applying PCA to our reshaped array reduces our channel dimension *C* to our predetermined number of principal components *K* followed by a reshaping of our data into (*H*, *W*, *K*), which preserves our spatial dimensions. The number of principals *K* was determined via empirical performance in our specific application with the aim of balancing between achieving higher performance metrics while keeping computational complexity low.

The final step is to convert each three-dimensional tensor into a four-dimensional tensor. Here, it is necessary to apply 3D CNNs as they typically deal with volumetric data as they expect the input shape to be (*N*, *H*, *W*, *D*, *C*) [86], where *N* is the batch size and *D* is the new depth dimension that represents the number of channels in our image. In this case, this will correspond to *K,* specified above.

#### 2.11.2. Model Architecture

The protein content estimation task consisted of two phases. The first was based on regression modeling using acquired truth labels representing the percentage of seed mass which was taken up by proteins. The second was classification using the three classes mentioned earlier in this paper as either low, medium, or high depending on the criteria specified.

**Figure 10:**
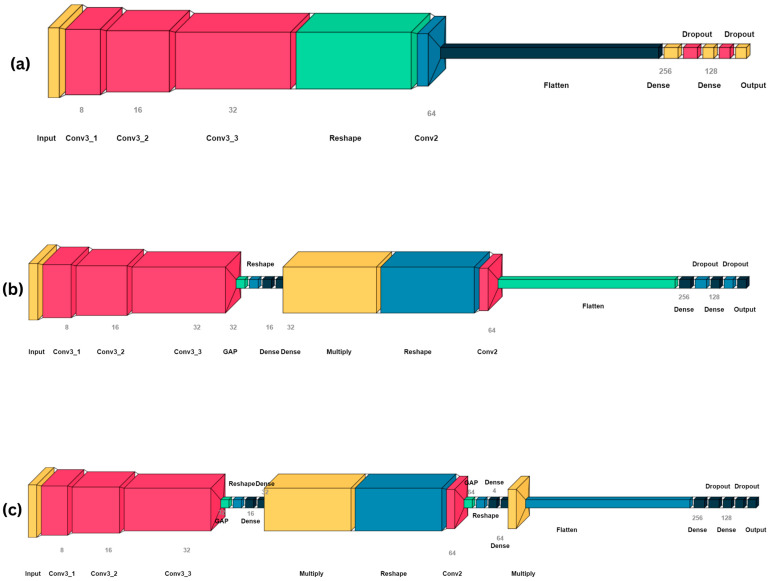
Visualization of the architectures of the different variations of HybridSN used in this study, including (**a**) the original architecture, (**b**) the modified architecture with global attention, and (**c**) the final modified model architecture with an additional attention mechanism of the Squeeze-and-Excitation network.

For image-based protein content estimation, we used a modified version of HybridSN [87], a hybrid convolutional neural network (CNN) that combines the capabilities of 3D convolution layers which have typically seen use with 3D imagery such as 3D lidar (Voxel R-CNN) and the 2D convolutional layers used for machine learning with the standard 2D imagery for classification, segmentation, and regression tasks. This architecture is summarized in Figure 10a. The nature of this structure allows the model to leverage the depth of spectral analysis provided by the 3D convolution layers and the spatial detail recognition capabilities of the 2D convolution layers.

Assuming that our individual HSI data cubes can be denoted by $Xi\in \;R^{\left(H\times W\right)\times D}$, with D as the number of bands, then for each input $X_{i}$, a corresponding real-valued number $y_{i}\in R$ represents the target variable, which is represented as the percentage of the protein content’s contribution to the overall mass of a seed.

The 3D convolution layer applies a 3D filter across the spatial and spectral dimensions of our input image, obtaining a dot product of kernel weights and local regions of the input volume covered and producing a 3D feature map in an operation that can be summarized by the following expression:

(10) $$F(x,y,z)=\sum_{i=0}^{a-1}\sum_{j=0}^{b-1}\sum_{k=0}^{c-1}V(x+i,\;y+j,\;z+k)\cdot K(i,j,k)$$

assuming a kernel of size a×b×c moving over input volume V at position (x,y,z) on the output feature map.

This approach ensures that we capture both spatial and spectral feature information of our input hypercube, and by stacking multiple 3D convolution layers, the model can learn a hierarchy of features, from the simplest patterns in the earlier layers to more complex features in the deeper layers.

This can be further condensed into

(11) $$F_{l}=\sigma (B_{l}+\sum_{i,j,k}X_{i,j,k}*K_{l})$$

where $F_{l}$, $B_{l}$, and $K_{l}$ represent the feature map, bias term, and the 3D kernel, respectively, at the l-th layer, and σ is the activation function.

The 3D convolutions are subsequently followed by a 2D convolution operation as shown below:

(12) $$F_{m}^{\prime }=\sigma (B_{m}^{\prime }+\sum_{i,j}F_{Lij}^{\prime }*K_{m}^{\prime })$$

with $F_{Lij}^{\prime }$ being the output feature map from the previous 3D convolution layers. Finally, we flatten the feature map into a vector and pass through a vector $V\epsilon \;R^{n}$, which is passed through a series of dense layers, with the final output being

(13) $$D_{p}=\sigma (W_{p}\cdot D_{p-1}+b_{p})$$

where $D_{p}$, $W_{p}$, and $b_{p}$ are the output, weight, and bias, respectively, of the p-th layer, and $D_{p-1}$ is the output from the previous dense layer or the flattened vector $V\epsilon \;R^{n}$ in the case of the first dense layer. The final regression output thus becomes

(14) $$Y=W_{k}\cdot D_{k-1}+b_{k}$$

With Y being the predicted continuous values corresponding to our regression targets. To train the regression model, we used the mean squared error (MSE) loss function, which minimizes the average squared difference between the predicted and true protein content values, ensuring accurate predictions of the continuous target variable.

As for the classification task, where if we assume the same notations for our input vectors as before, for each Xi, there exists a corresponding $y_{i}=\left[y_{i,1},y_{i,2},\ldots ,\;y_{i,C}\right],$ such that $y_{i,j}\in \;\left\{0,1\right\},\;\sum_{j=1}^{C}y_{ij}=1,\;\mathrm{and}\;C=3$, which corresponds to our number of protein content classes.

Consequently, our final output becomes

(15) $$Y={softmax(W}_{k}\cdot D_{k-1}+b_{k})$$

where Y is the probability distribution over classes. To train this classification model, we used the categorical cross-entropy loss function, which measures the divergence between the predicted class probabilities and the true one-hot encoded labels.

In the HybridSN documentation [87], the authors noted that 25 × 25 spatial dimensions worked best for their application. For our case, we also experimented with spatial dimensions of 32 × 32 and found that they produced the best results for our dataset. This resulted in an input size of *n* × 32 × 32 × 662 for our model. We also experimented with different kernel sizes for the different 3D layers and documented the results. The first set of kernel sizes was the original kernel size specified from layer 1 to layer 3 as 3 × 3 × 7, 3 × 3 × 5, and 3 × 3 × 3. The second set was 3 × 3 × 11, 3 × 3 × 9, and 3 × 3 × 7 and the third set was 5 × 5 × 15, 5 × 5 × 11, and 5 × 5 × 9. We also experimented with different combinations of batch sizes and learning rates. The empirical performances of each combination set were then documented.

For validation of the models, we calculated the errors and accuracies based on several metrics. The first was the coefficient of determination, calculated as

(16) $$R^{2}=1-\frac{\sum_{i=1}^{n}{\left(y_{i}-{\hat{y}}_{i}\right)}^{2}}{\sum_{i=1}^{n}{\left(y_{i}-{\bar{y}}_{i}\right)}^{2}}$$

where $y_{i}$ is the observed values, $\hat{y}$ is the predicted values, $\bar{y}$ is the mean of the observed values, and n is the number of observations.

We also evaluated the model using mean absolute error, which calculates the average prediction error in the units of variable being predicted and is denoted as

(17) $$MAE=\frac{1}{n}\sum_{i=1}^{n}\left|y_{i}-{\hat{y}}_{i}\right|$$

Mean squared error is more useful for detecting outliers, as it penalizes larger errors more severely than it does smaller ones, and is calculated as

(18) $$MSE=\frac{1}{n}\sum_{i=1}^{n}{\left(y_{i}-{\hat{y}}_{i}\right)}^{2}$$

Meanwhile, root mean square provides the standard deviation of the residuals (prediction errors), where lower values indicate a better fit, and is calculated simply as the square root of the MSE:

(19) $$RMSE=\sqrt{MSE}$$

For the classification task, we used four different metrics: accuracy, precision, recall, and F1 score. Accuracy measures the overall correctness of the model and works well when the classes are balanced. It is defined as

(20) $$Accuracy=\frac{True\;Positives\;\left(TP\right)+True\;Negatives\;(TN)}{Total\;Number\;of\;Samples}$$

Precision is a measure of accuracy of positive predictions and is useful when the cost of a false positive is high. It is defined as

(21) $$Precision=\frac{True\;Positives\;\left(TP\right)}{True\;Positives\;\left(TP\right)+False\;Positives\;\left(FP\right)}$$

Recall measures the model’s ability to detect all relevant cases within a dataset and is used then the cost of a false negative is high. It is defined as

(22) $$Recall=\frac{True\;Positives\;\left(TP\right)}{True\;Positives\;\left(TP\right)+False\;Negatives\;\left(FN\right)}$$

Finally, F1 score is the harmonic mean of precision and recall and can be expressed as

(23) $$F1=2\times \frac{Precision\times Recall}{Precision+Recall}$$

The HybridSN model was modified through the addition of an attention mechanism to process the feature map output of the 3D convolution block. The global attention block [88] calculates the importance weights of each channel in the feature map. These weights are then used to scale the original channel values, effectively amplifying important channels and attenuating the less relevant ones.

A Global Average Pooling layer takes input tensor I with dimensions H×W×D×C, with H,W, D, and C as the height, width, spectral depth, and number of channels from the immediately preceding 3D convolution layer, reducing I to a 1D tensor with an equal number of elements to the number of channels, C. A dense layer applies a reduction in dimensionality followed by ReLU activation, and a second layer projects back to the original number of channels with a sigmoid activation to create attention map A:

(24) $$A=\sigma (W_{2}\cdot {(ReLU(W}_{1}\cdot G+b_{1}))+b_{2})$$

where $W_{1}\in R^{C\times \frac{C}{2}}$ and $b_{1}\in R^{\frac{C}{2}}$ are the weight matrix and bias of the first layer, $W_{2}\in R^{\frac{C}{2}\times C},\;\mathrm{and}\;b_{2}\in R^{C}$ are the weight matrix and bias of the second layer, and our attention map has dimensions of 1×1×1×C.

The final operation involves scaling each channel of the input feature by its corresponding attention weight:

(25) O=I⊙A

Here, O retains the dimensions H×W×D×C.

In essence, the attention mechanism works by learning a set of weights from the input tensor itself, which is then applied to modulate the input tensor’s channels, allowing the network to prioritize some channels over others. The final architecture is summarized in Figure 10b.

An additional architectural modification was implemented on the HybridSN network through the addition of a Squeeze-and-Excitation network after the 2D convolution layer to recalibrate channel-wise feature importances. First introduced by Jie [89], the Squeeze-And-Excitation block involves initially compressing each feature map into a single number by averaging over all the spatial dimensions, producing a vector of size equal to the number of channels. Given dimensions H×W×C of our feature map, this can be expressed as

(26) $$z_{c\;}=\frac{1}{H\times W}\sum_{i=1}^{H}\sum_{j=1}^{W}F_{ijc}$$

This is followed by a bottleneck layer which reduces the dimensionality of the output from GPA, compressing channel-wise features to a lower-dimensional representation, and an expansion layer which reverses the representation back to its original channel-wise representation, going through a sigmoid transformation first. The two can be expressed as follows:

(27) $$v={ReLU(W}_{1}\cdot z+b_{1})$$

(28) $$s={Sigmoid(W}_{2}\cdot v+b_{2})$$

Finally, channel-wise multiplication is applied and used to scale the original feature map *F*:

(29) $$F^{\prime }=s⊙F$$

## 3. Results and Discussion

### 3.1. Co-Registration Results

**Figure 11:**
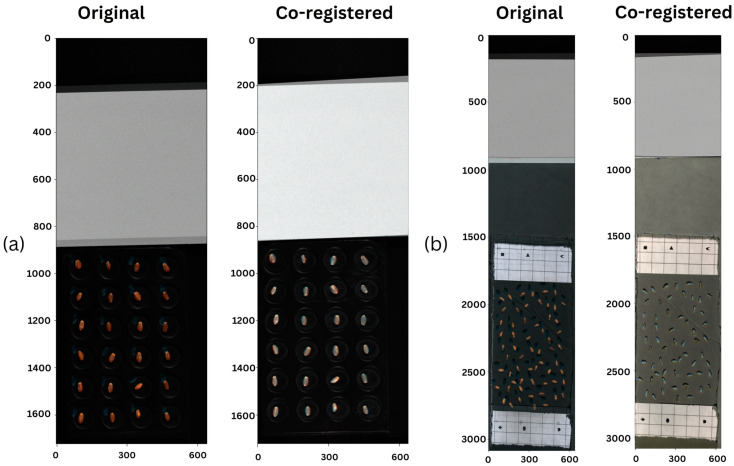
Analysis of the visual differences between simple resampling strategy and our semi-automated method to co-register VNIR and SWIR scans for (**a**) the first batch of scans and (**b**) the second batch of scans.

As explained in our methodology, co-registration was a necessary and crucial step in the pipeline that made it possible to combine the full range of bands from VNIR and SWIR together and thus create a new method to determine protein content in wheat seeds. As such, the goal was to minimize errors as much as possible to ensure we had correctly aligned multi-sensor seed scans. We compared the errors of stacking the images using a simple resampling approach against the errors after using our method. The results are summarized in Table 4 and illustrated in Figure 11.

Overall, our method produced significantly less errors compared to the more simplified resampling method.

### 3.2. Protein Content Estimation

#### 3.2.1. Feature-Based Machine Learning for Protein Content Estimation

The best overall model was Random Forest, with an R^2^ of 0.77, comparable to when Caporaso et al. [11,15] used a standard normal variate and first derivatives as features. In our case, we found that the model trained on ratios of all the bands fared better than the ones trained on ratios of the most informative bands alone, with a coefficient of determination score of 0.77 and 0.75 for Random Forest for bottom and top orientations, respectively, and 0.66 and 0.67 with SVR for bottom and top orientations, respectively. This could be an indication that even though some wavelengths, individually, are not as discriminative for seed protein estimation as those in the NIR region, they still contribute to the model training if their information is extracted and processed in a different way, rather than used directly. The results of these experiments are summarized in Supplementary Table S1.

**Figure 12:**
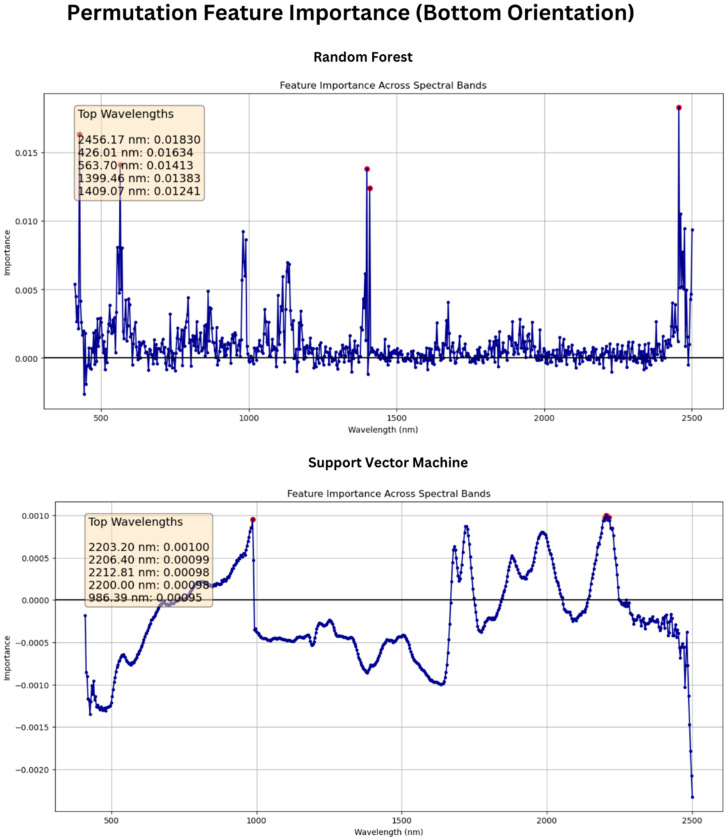
Permutation feature importance analysis for wheat seeds with crease facing up.

**Figure 13:**
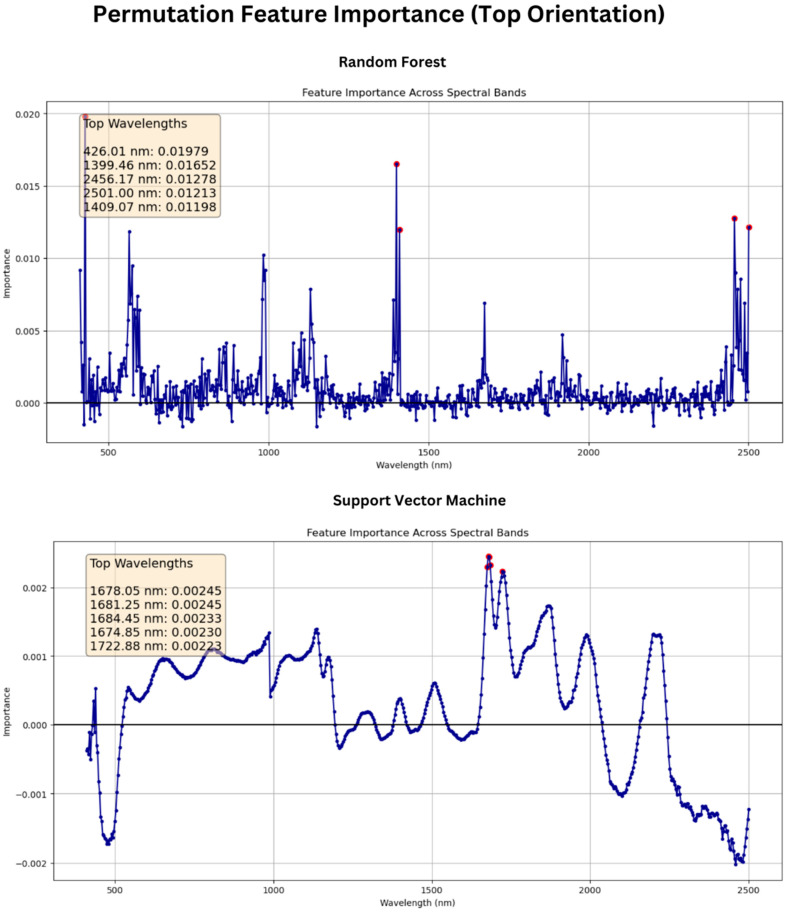
Permutation feature importance analysis for wheat seeds with crease facing down.

Feature permutation analysis indicated that the wavelengths 2456 nm, 426 nm, 564 nm, 1399 nm, and 1409 nm appeared to be the most important input features for Random Forest modeling for the bottom side of the wheat scans and that 426 nm, 1399 nm, 2456 nm, 2501 nm, and 1409 nm were most important for top orientation, as seen in Figure 12. Meanwhile, the best wavelengths for SVR were 2203 nm, 2206 nm, 2212 nm, 2200 nm, and 986 nm for the bottom orientation and 1678 nm, 1681 nm, 1684 nm, 1674 nm, and 1722 nm for top orientation, as shown in Figure 13. These findings suggest the dominance of data in the NIR region over those of the visible region of the spectrum for studying the chemical characteristics of wheat seeds and corroborate the findings of previous research.

#### 3.2.2. Image-Based Machine Learning for Protein Content Estimation

For image-based modeling, we found the best model to have an R^2^ of 0.70 for the ventral side (bottom orientation) and 0.65 for the dorsal side (top orientation), with comparably similar performance across the different kernel size sets and model types. These are summarized in Supplementary Tables S2 and S3.

However, when compared with fixed values of batch size and kernel sizes, we found that the addition of attention would improve the performance of the model significantly and reduce errors, as indicated in Supplementary Tables S4 and S5 for the performance of the bottom-oriented and top-oriented seeds, respectively.

Increasing the kernel depth translated to a better performance of the model in some cases, with the best overall model working well with the second kernel size set, but this was not always the case. We found that increasing the spatial dimensions of the kernel to 5 × 5 made the performance of the model much worse before we ceased experimenting with higher spatial kernel dimensions.

**Figure 14:**
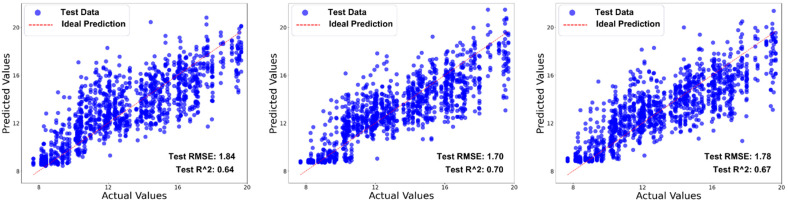
Scatterplot of predictions for seed protein content using the different variations of HybridSN. From left to right: original HybridSN architecture, HybridSN with global attention block, and HybridSN with global attention and Squeeze-and-Excite network blocks. The tighter clustering of predicted values around the ideal prediction line for the second model suggests better predictions with less variance for that model.

A cross-examination of the models using scatterplots, as shown in Figure 14, demonstrates that the original model struggles with instances on all ends and those in the middle, and while our models with attention achieve a slight improvement with the middle-placed values, they still also struggle with instance on the ends, especially the higher ends. This observation was made on both the training and test datasets. Overall, all models were able to achieve an RMSE of less than 2%.

These results demonstrate that our CNN model can produce results that match or improve upon the performance of feature-based models, producing R^2^ scores at least similar to or higher (0.65 and 0.70) than those produced by SVR (0.65 and 0.66) in experiments while being outperformed by the best feature-based model, Random Forest, by no more than 9%. This is encouraging because developing end-to-end, rapid image-based AI-powered approaches is a prerequisite for determining seed composition in operational settings.

The inclusion of the 3D CNN block in the HybridSN architecture allowed the model to learn and retain valuable spectral features from the HSI, in addition to the spatial features. This suggests that relying solely on spatial features from seed scans may be insufficient, and that retaining spectral information is crucial for accurate prediction of protein content.

Furthermore, the inclusion of a global attention layer after the 3D CNN block and a Squeeze-and-Excitation block improved the model’s performance. The global attention layer allows the model to focus on the most relevant spatial–spectral features across the entire input, while the SE network adaptively recalibrates channel-wise feature responses in the spatial domain.

The performance gains achieved by incorporating these attention mechanisms suggest that the model benefits from selectively emphasizing informative spectral and spatial features. Notably, the global attention layer, which operates on the spectral dimension, likely contributes to the model’s ability to better utilize the spectral information present in the HSI data. This further supports the argument that spectral features play a crucial role in accurately predicting protein content from wheat seed HSI scans.

When classifying the wheat samples according to protein content in the three classes, we found that our models were able to more easily discriminate between the different wheat seed samples based on protein content, with a high accuracy of 0.82 for both orientations on our test dataset. Additionally, we found high precision, recall, and F1 scores for most of the models, indicating the well-balanced performance of the models in identifying true positives while minimizing false positives. These results are available in Supplementary Tables S6 and S7. However, we found that the addition of attention mechanisms did not improve results enough to justify their use and in some cases affected the performance negatively across all metrics. This appears in line with findings from another study using global attention [90].

For our study, this could be because the increased complexity of using attention mechanisms may have led to further overfitting, especially since our dataset was small (about 5000 per orientation). These mechanisms inherently add more parameters and layers to the model, and this often requires substantial data to help the models generalize more.

Although feature-based approaches that utilized spectral data (indies optimized for extracting features of interest) provided good results, they require manual selection of indices and feature engineering, which are often challenging without subject matter expertise. Spatial and texture information of seed kernels, which has been proven to be important in previous studies, is not considered in feature-based approaches. The image-based approaches proposed in this paper leverage both spatial and spectral features from hyperspectral images and can be implemented in automated seed sorting and breeding processes.

### 3.3. Sources of Error

The rich information contained in full-spectrum HSI data necessitates a discussion on the possible contemporary use of the technology in real-world applications. However, before this idea can be realized, we need to address and nullify the effects of errors that impact negatively on the performance of our models. A few sources we identified include the following:

- The optical and surface topography properties of wheat seeds potentially play a big role in the performance of the model, as we observed in our own results and others [91]. The top sides of the wheat seeds (crease-down) were observed to have a smoother surface with higher reflectivity scores than those of the bottom orientation of the same seed. The bottom side of the seeds has an uneven surface caused by the crease, which can result in scattering and incident reflection. A switch to using absorbance data, like Caporaso et al. [11], or harmonization of both can provide more insight into how much impact these effects have on the final output of our modeling efforts and result in better performances.
- We also theorize that the sample size affected the accuracy of our predictive models. Here, this limitation manifests in two ways. One is the actual number of wheat samples that were scanned and assessed for protein content via combustion analysis. We had a total of nearly 10,000 samples to use for training, which limited us from experimenting with advanced models such as transformers, which require massive datasets for training [92,93]. Secondly, we acquired our ground-truth protein content using Dumas combustion, which has a minimum weight limit of samples in order to produce protein content labels. Consequently, the protein content values were acquired per sample of at least 60 wheat kernels rather than per individual wheat kernel. It is plausible that this disparity in granularity between the training data and the corresponding ground-truth labels prevented a more precise mapping of the spatial–spectral characteristics of wheat seeds with protein content.

### 3.4. Future Direction

The main challenge of using HSI for single-wheat-kernel prediction of protein is the large sizes of the datasets. The average file size of a single scanning session was easily between 36 GB and 72 GB. Handling such large datasets requires a large amount of storage space and computing power and can prove to be challenging for most research efforts. One possible solution for this is to use spectral and spatial binning while performing image correction during the processing stage. Binning involves downsampling the spatial or spectral properties of our image scans to a specified degree and will reduce the file sizes significantly at the cost of losing some information.

Secondly, to the best of our knowledge, no commercial tools exist that can co-register VNIR and SWIR images collected with bench-top scanners at the time of writing. This can prove to be cumbersome when you must process hundreds of datasets. We developed a semi-automated co-registration tool that allowed for batch processing of seed scans, resulting in faster processing of huge datasets.

Future research and development should focus on the scalability of the co-registration and image calibration process by introducing an accurate, fully automatic co-registration workflow. Additionally, foundational models need to be developed with increased sample data for training large language models so that the methods developed in this paper can be deployed in industrial settings.

## 4. Conclusions

The objectives of this paper were to (1) develop robust methods for seed trait prediction using a bench-top hyperspectral imaging scanner in the VNIR and SWIR region; (2) determine optimal wavelengths that are useful for the prediction of seed traits such as protein content; and (3) investigate how the orientation of wheat kernels during scanning affected the viability and usefulness of the data for predictive modeling.

Our results show the following:

1. The end-to-end CNN with attention mechanisms explained wheat kernel protein content with up to 0.7 R^2^ values. R^2^ values were 0.70 and 0.65 for ventral and dorsal seed orientations, respectively, indicating that seed orientation for scanning has a significant impact on the predictive power of CNN models.
2. Consistent with many previous studies, feature-based RF outperformed the direct imagery-based CNN with a higher R^2 of 0.77. However, feature engineering requires expert knowledge of the features (spectral indices in our case) and is time-consuming, not being scalable for operational scenarios. End-to-end prediction capabilities with CNNs are preferred for the automation of wheat protein estimation for breeding.
3. The CNN model predicted low, medium, and high protein concentrations with an R^2 of 0.82. The finding is significant because this type of classification provides more meaningful means (compared to estimating the exact amount of protein content) for crop breeding and seed profiling. Classification-based seed profiling with hyperspectral imaging and machine learning techniques plays a pivotal role in advancing precision breeding practices, optimizing seed sorting processes, and enabling targeted agricultural input applications.
4. Sensitivity analysis showed that NIR bands outperform all other hyperspectral bands for seed protein content estimation and have higher importance scores for models. However, when combined with information from other bands in the hyperspectral range, it was possible to produce more robust models.

Our study highlights significant potential for advancing agricultural practices in a targeted and efficient way. By moving beyond traditional NIR spectroscopy and incorporating a broader spectrum of data, we can deepen our understanding of factors that influence seed quality, with far-reaching implications including the following:

1. Enabling more precise breeding practices by enabling breeders to accurately and rapidly predict desirable qualities of seeds, with the knock-on effect of improved yield and higher quality crops.
2. Additionally, this technology could also lead to a more targeted use of agricultural inputs, such as fertilizers and pesticides, based on the genetic information of the seed material, as identified by our model.
3. Further, the orientation-dependent findings in our study suggest that a customized approach to agricultural machinery and processes is necessitated and should be geared towards optimizing seed handling in a manner that ensures high accuracy.

## Figures and Tables

**Table 1:** Number of instances for each genotype class.

Class	B1	B9	M	MT	N	NT	W	WB	WBT
Frequency	384	94	207	422	373	118	304	239	257

**Table 2:** Ground-truth statistics.

Orientation	Central Tendency			Spread				Position		Shape	
	Mean	Median	Mode	Range	Variance	Standard Deviation	IQR	Q1	Q3	Skewness	Kurtosis
Bottom	13.22978	12.81	10.44	11.96	9.586461	3.094586	5.36	10.63	15.99	0.200924	−0.95656
Top	13.48322	13.06	10.44	11.96	8.773358	2.961986	5.12	10.99	16.11	0.212506	−0.95651

**Table 3:** Summary of datasets generated.

Dataset Number	1	2	3
Dataset Type	All spectral bands	Top 100 spectral bands + band ratios of top 100 bands	All spectral bands + ratios of all bands

**Table 4:** Comparison of errors of co-registration when using simple resampling methodology vs. using our custom methodology.

	Average RMSE	Max RMSE	Min RMSE	Median RMSE
Resampling	0.1246	0.8846	0.0804	0.1211
Our method	0.0487	0.1381	0.0094	0.0205

## Data Availability

Data are not available for public release.

## Supplementary Materials

The following supporting information can be downloaded at https://www.mdpi.com/article/10.3390/s25020303/s1: Table S1: Best performing ML models for protein content regression; Table S2: Best performing deep learning models for protein content regression (bottom orientation); Table S3: Best performing deep learning models for protein content regression (top orientation); Table S4: Comparison of effects of different hyperparameter combinations on final coefficient of determination scores and reported errors for the different CNN architectures (bottom orientation); Table S5: Comparison of effects of different hyperparameter combinations on final coefficient of determination scores and reported errors for the different CNN architectures (top orientation); Table S6: Evaluation of protein content classification models for the different CNN architectures and different combinations of hyperparameters (bottom orientation); Table S7: Evaluation of protein content classification models for the different CNN architectures and different combinations of hyperparameters (top orientation).
